# Patch tests in patients using immunosuppressants and/or cytokine inhibitors: descriptive analysis of 16 cases^☆^

**DOI:** 10.1016/j.abd.2022.03.005

**Published:** 2022-11-24

**Authors:** Rosana Lazzarini, Nathalia T. Kawakami, Nathalie Suzuki, Mariana de Figueiredo da Silva Hafner

**Affiliations:** a Dermatology Clinic, Santa Casa de São Paulo, São Paulo, SP, Brazil; b Faculty of Medical Sciences, Santa Casa de São Paulo, São Paulo, SP, Brazil

Dear Editor,

Patch tests are the reference proof for the etiological diagnosis of *allergic contact dermatitis* (ACD), related to the delayed type 4 hypersensitivity reaction (Gell & Coombs).^1^

The use of drugs that interfere with patient immune response, such as corticosteroids, cyclosporine, methotrexate, azathioprine, mycophenolate mofetil, and the latest cytokine inhibitors (infliximab, adalimumab), could be considered a limitation to undergo patch tests since they act by inhibiting cell responses. On the other hand, the use of these medications has become increasingly common and, often, their withdrawal is not possible due to the underlying disease. Moreover, studies show that many patients are capable of developing eczematous reactions even when using these drugs.^2^, ^3^, ^4^, ^5^

The present study aimed to investigate suspected cases of ACD submitted to patch testing in a non-ideal situation (patients who were receiving immunosuppressive drugs and/or cytokine inhibitors).

Data from 16 patients tested between 2009 and 2021 and who were using any of the abovementioned medications at the time of the test were retrospectively analyzed. Different series of allergens were used, the indications of which were based on anamnesis and clinical picture.

The tests were applied to the upper back region and removed after 48 hours. The results were obtained after 48 and 96 hours. The possible reactions were: negative, weakly positive (1+) (erythema, infiltration or papules); strongly positive (2+) (edema and/or vesicles); and very strongly positive (3+) (bullae or ulceration).

The mean age of the patients was 49 years, consisting of 12 women and four men. The median time of the dermatitis was 37 months (3‒180 months). The medications used by the patients at the time of the tests were: prednisone in nine cases, methotrexate in seven, azathioprine in four and infliximab, cyclosporine, cyclophosphamide, tacrolimus and adalimumab in one case each. Some patients used more than one medication at the same time. The doses used by the patients varied according to the indication and the disease stage. In the case of prednisone, they varied between 5 and 40 mg per day, and for methotrexate, between 10 and 15 mg per week. Regarding cyclophosphamide, the patient had been submitted to pulse therapy one month before the test with a dose of 1 g, and azathioprine was used at doses of 100 and 150 mg/day.

The reasons for using these medications were difficult-to-control eczema (four cases), collagen diseases (systemic and discoid lupus, Behçet's disease and antisynthetase syndrome) in eight cases, Crohn's disease, Cushing's syndrome and psoriasis in one case each.

Among the tested patients, ten (62.5%) had at least one positive test and six (37.5%) had all negative results. One of the cases with a negative initial test was positive in a new test performed after drug withdrawal (methotrexate), a result that was previously relevant. In the new test, positivity was observed for paraphenylenediamine (PPDA); contact and ACD had occurred after the application of a temporary tattoo (“henna”) in adolescence.

After completion of the tests, nine (56.3%) cases were considered to have a final diagnosis of ACD. Other diagnoses were psoriasis, irritant contact dermatitis, atopic dermatitis, drug reaction and dyshidrosis with one case each. Two cases remained without a definitive diagnosis, under outpatient follow-up. Table 1 shows the results and intensities of the patch tests and the final diagnoses in the 16 assessed patients.

**Table 1:** Distribution of the patch test results and final diagnoses in relation to medications used by the patients undergoing patch testing.

Patient	Sex	Age	Medication	Dose	Indication	Patch test	Final diagnosis
1	F	52	Infliximabe; azatioprina	5 mg/kg; 125 mg	Chron’s disease	Balsam of Peru (1+), PPDA (3+)	Hair dye ACD
2	F	75	MTX	15 mg/wk	Photosensitivity	Negative	Photosensitivity a/e
3	F	50	Prednisone	5 mg/d	Cushing’s disease	Thimerosal (1+), Kathon CG (1+), MI (2+), Formaldehyde (1+)	Hands ACD
4	M	64	Cyclosporine + MTX	75 mg/d; 15 mg/wk.	SLE	Negative	Atopic dermatitis
5	F	56	MTX	15 mg/wk.	SLE	Potassium bichromate (1+), nickel sulfate (1+)	Metals ACD
6	F	44	Prednisone	10 mg/d	SLE	Negative	Inverted psoriasis
7	f	42	Prednisone	5 mg/d	Behçet disease	Negative	Drug reaction
8	F	56	Prednisone	10 mg/d	DLE	PPDA (3+), 2-nitro-PPDA (2+), *m*-aminophenol (2+), *p*-aminophenol (2+)	Hair dye
9	F	40	Prednisone + MTX	10 mg/d; 15 mg/wk.	SLE	PCMX (1+), *m*-aminophenol (1+), ammonium persulfate (1+)	Unknown relevance
10	M	24	MTX	15 mg/wk.	Dyshidrotic eczema of the hands	Negative	Dyshidrosis^a^
11	F	29	Azathioprine; prednisone	125/d; 5 mg/d	SLE	Cobalt chloride (1+), nickel sulfate (1+), TSFR (1+)	Nail polish + metals ACD
12	F	60	MTX	10 mg/wk.	Eczema	Neomycin (1+); Amerchol (2+)	Topical medication + cosmetics ACD
13	F	57	Prednisone + cyclophosphamide	40 mg/d; pulse 1 g	Antisynthetase Sd. + Sjogren	FM 2 (1+), Lyral (1+)	Fragrance ACD
14	F	51	Azathioprine + Prednisone + MTX	150 mg/d; 50 mg/d; 20 mg/wk.	SLE	PPDA (2+); FM 1(1+); FM 2 (1+)	Hair dye + fragrances ACD
15	F	52	MTX + Adalimumab	10 mg/wk.	Psoriasis	Nickel (2+); Sodium tetrachloropalladate (2+)	Metals + psoriasis ACD
16	M	33	Prednisone + Azathioprine + tacrolimus	5 mg/d; 100 mg/d; 4 mg/ d	Kidney transplant	Negative	Irritative CD of the hands

MTX, Methotrexate; SLE, systemic lupus erythematosus; DLE, discoid lupus erythematosus; ACD, allergic contact dermatitis; Kathon CG, methylisothiazolinone + methylchloroisothiazolinone; MI, methylisothiazolinone; PPDA, paraphenylenediamine; PCMX, chloroxylenol; TSFR, toluene sulfonamide formaldehyde resin; FM 2, fragrance mix 2 (lyral, citral, geraniol, farnesol, citronellol, cinnamic hexyl aldehyde, coumarin); FM 1, fragrance mix 1 (amyl cinnamal, cinnamal, cinnamic alcohol, eugenol, oak moss absolute, geraniol, hydroxycitronellal and isoeugenol).

a Positive test for paraphenylenediamine (PPDA) after retesting without medication.

Some studies show that the use of corticosteroids (especially >30 mg/day) causes partial to complete suppression of the test results in many cases; however, some patients may maintain positive reactions, as observed in one of the cases, which tested positive for fragrance even during the use of 40 mg/day of prednisone.^3^

Studies published in the literature show positive results in tested patients who were using different immunosuppressants or modulators with variable results. Kim et al., in 2014, published a series of cases showing that biologicals would not interfere with test results, although many of these medications have shown an action on cytokines common to psoriasis and ACD (TNF-a; IL-23 and IL-17). In the same year, Wentworth et al. published a small case series of patients receiving methotrexate and/or mycophenolate mofetil with positive and relevant tests.^2^, ^3^

Immunosuppressants affect the first phase of the immune response in different ways: by blocking IL-1, IL-2, TNF-a, and granulocyte colony-stimulating factor (corticosteroids); by reducing the number of Langerhans cells, and weakening the activity of T cells (azathioprine); by affecting DNA synthesis, decreasing IL-1 activity and TNF-a release (methotrexate); by reducing T-cell proliferation, blocking dendritic cell function, and limiting stimulation of cytokine release (mycophenolate mofetil).^3^ However, studies have suggested that concentrations in the test application area seem to be high enough to overcome the suppressive capacity of these medications.^4^, ^5^

The main limitations of the study comprised the low number of analyzed patients, and the use of drugs of different classes and in different doses. However, the current small series of cases showed that patients using immunosuppressants obtained positive and relevant results, indicating that it’s possible to perform them, although a careful interpretation of the results is necessary. When testing under these conditions, the lowest possible dose of medication should be used and consider retesting after therapy discontinuation. Studies with larger populations, with careful follow-up of patients, are necessary for further clarification on the subject.

## Financial support

None declared.

## Authors' contributions

Rosana Lazzarini: Project of the study, analysis and interpretation of data; intellectual participation in the propaedeutics and therapeutic conduct of the cases; critical review of the manuscript; final approval of the manuscript.

Nathalia T. Kawakami: Collection, analysis and interpretation of data; literature review; approval of the final version of the manuscript.

Nathalie Suzuki: Analysis and interpretation of data; intellectual participation in the propaedeutics and therapeutic conduct of the cases; critical review of the manuscript; approval of the final version of the manuscript.

Mariana de Figueiredo da Silva Hafner: Analysis and interpretation of data; intellectual participation in the propaedeutics and therapeutic conduct of the cases; critical review of the manuscript; approval of the final version of the manuscript.

## Conflicts of interest

None declared.
